# Genomic sequencing identifies a few mutations driving the independent origin of primary liver tumors in a chronic hepatitis murine model

**DOI:** 10.1371/journal.pone.0187551

**Published:** 2017-11-08

**Authors:** Zuyu Yang, Mingming Jia, Guojing Liu, Huaining Hao, Li Chen, Guanghao Li, Sixue Liu, Yawei Li, Chung-I Wu, Xuemei Lu, Shengdian Wang

**Affiliations:** 1 CAS Key Laboratory of Genomics and Precision Medicine, China Gastrointestinal Cancer Research Center, Beijing Institute of Genomics, Chinese Academy of Sciences, Beijing, China; 2 Key Laboratory of Infection and Immunity, Institute of Biophysics, Chinese Academy of Sciences, Beijing, China, University of Chinese Academy of Sciences, Beijing, China; 3 State Key Laboratory of Biocontrol, College of Ecology and Evolution, Sun Yat-Sen University, Guangzhou, China; 4 Department of Ecology and Evolution, University of Chicago, Chicago, Illinois, United States of America; University of Hong Kong, HONG KONG

## Abstract

With the development of high-throughput genomic analysis, sequencing a mouse primary cancer model provides a new opportunity to understand fundamental mechanisms of tumorigenesis and progression. Here, we characterized the genomic variations in a hepatitis-related primary hepatocellular carcinoma (HCC) mouse model. A total of 12 tumor sections and four adjacent non-tumor tissues from four mice were used for whole exome and/or whole genome sequencing and validation of genotyping. The functions of the mutated genes in tumorigenesis were studied by analyzing their mutation frequency and expression in clinical HCC samples. A total of 46 single nucleotide variations (SNVs) were detected within coding regions. All SNVs were only validated in the sequencing samples, except the *Hras* mutation, which was shared by three tumors in the M1 mouse. However, the mutated allele frequency varied from high (0.4) to low (0.1), and low frequency (0.1–0.2) mutations existed in almost every tumor. Together with a diploid karyotype and an equal distribution pattern of these SNVs within the tumor, these results suggest the existence of subclones within tumors. A total of 26 mutated genes were mapped to 17 terms describing different molecular and cellular functions. All 41 human homologs of the mutated genes were mutated in the clinical samples, and some mutations were associated with clinical outcomes, suggesting a high probability of cancer driver genes in the spontaneous tumors of the mouse model. Genomic sequencing shows that a few mutations can drive the independent origin of primary liver tumors and reveals high heterogeneity among tumors in the early stage of hepatitis-related primary hepatocellular carcinoma.

## Introduction

Hepatocellular carcinoma (HCC), one of the leading causes of cancer-related death worldwide, is characterized by phenotypic and molecular heterogeneity related to various etiologies. More than 90% of HCCs arise in the context of chronic hepatitis and cirrhosis[1]. Long-term chronic inflammation causes oxidative damage, DNA mutations and metabolic stress, among other changes in the microenvironment, by releasing a variety of cytokines and chemokines; these alterations ultimately lead to cirrhosis. In cirrhosis, precancerous dysplastic lesions transform into early well-differentiated HCCs that progress into progressed HCCs and then advanced HCCs. Several studies using whole-genome and whole-exome analysis have been performed on human HCCs to provide a comprehensive understanding of genetic alterations, and these studies identified thousands or tens of thousands of somatic mutations[2,3], of which 4 to 362 are protein-changing somatic mutations, with an average number of 52.5 mutations per individual[3–6]. In addition, to confirm the previously known mutations in *TP53*, these studies also shed light on the importance of deregulation by somatic mutations of the *Wnt*-signaling components *CTNNB1* and *AXIN1*; chromatin regulators such as *ARID1A* and *ARID2*; amplifications of *MYC*, *FGF19* and *CCND1*; and HBV integration into the *TERT* and *MLL4* gene loci, which encode telomerase reverse transcriptase and histone lysine methyl transferase, respectively[7,8]. The number of non-silent mutations in protein-coding regions varies from study to study and among patients. Furthermore, the frequently altered genes discovered by these studies differ. The most striking observation is the distinct genetic alterations among HCC patients, even between synchronous multi-centric cancers[9,10] and within a single tumor[11]. By the time a tumor is clinically detected, individual tumor cells harbor numerous acquired mutations under selection (drivers) and an even greater number of events that offer no selective advantage (passengers). The genetic heterogeneity of HCC has complicated our understanding of the evolutionary process of tumors, and the key drivers of HCC tumorigenesis remain poorly understood.

Similar to natural speciation, tumorigenesis is a gradual evolutionary process involving the interaction of multiple genes and environmental components. After the proposal of the two-hit model of oncogenesis[12], and particularly after the discovery of the linear progression from benign polyps to colorectal cancer via a series of mutational events[13], tumorigenesis and progression were briefly envisioned as the result of a series of genetic variations that contribute selective advantages of proliferation and migration to tumor cells[14–16]. With the development of high-throughput sequencing technologies, cancer genomic variations have been identified from single nucleotide variations (SNVs), structural variations (SVs) to whole genome-doubling events[2–6,17–24]. Nevertheless, most cancer genomic studies are based on clinical samples, most of which were diagnosed to be highly malignant. Very early growing human tumors are difficult to detect, and whether any removed small tumor would have actually progressed is unknown. Thus, obtaining early-stage tumors and performing genomics sequencing could be very helpful for understating the population dynamics of tumor cells at an early stage, which may provide insight as to how to better prevent, detect and treat cancers.

In the past decades, mouse models have contributed significantly to our understanding of the molecular mechanisms underlying tumor initiation and progression [25] and have played an emerging role in the functional annotation of the complex cancer genome, such as in genomic studies of a mouse model of leukemia[26], medulloblastoma[27]and lung cancer[28]. Furthermore, a greater proportion of tumor drivers to passengers is expected in the mouse genome because the tumors can be formed in a short time period and the inbred mice share the same genetic background. We previously established a primary HCC mouse model in HBV transgenic mice by repetitive infusion of the anti-CD137 agonist mAb, which mimics the pathological process of human HCC developing from chronic hepatitis to liver cancer[29]. This mouse provides an ideal model to study early-stage tumor evolution. To better understand the genetic variations and identify potential tumor drivers of early-stage HCC, we use whole-exome sequencing (WES) and (or) whole genome sequencing (WGS) to characterize specific variations of tumors from this mouse HCC model.

## Results

### Sampling and sequencing of primary liver cancers developed from chronic hepatitis in HBV transgenic mice

**Fig 1:**
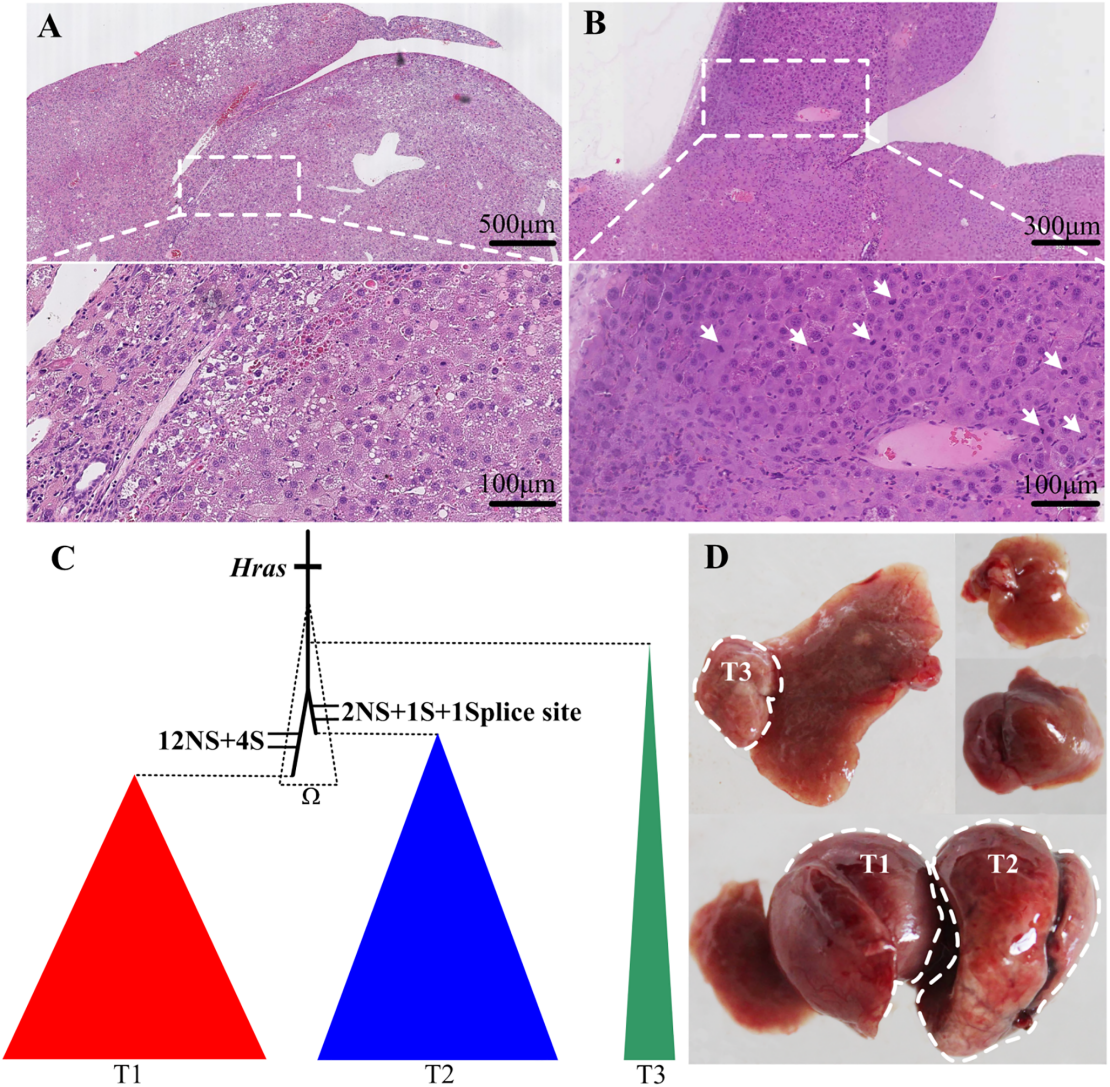
Pathological analysis of tumor tissues and phylogenetic relationships of the three tumors in the M1 mouse. HBV-transgenic mice were intraperitoneally injected with anti-CD137 Ab weekly up to five times. Live tumor nodules and adjacent non-tumor tissues were harvested at more than 11 months after the last injection. Representative tumor sections stained with H&E showing (A) hepatocellular adenomas (HCA), (B) hepatocellular carcinomas (HCC). Top: overview of tumor and pericancerous tissues. Bottom: zoomed-in view of the tumor in the top panel. White arrows indicate the uneven proliferation of hepatocytes. (C) Phylogeny of tumors from the M1 mouse based on validated SNVs. The ancestor lineage was defined as Ω. NS: nonsynonymous mutation, S: synonymous mutation. (D) Liver morphology and locations of 3 tumors in the liver of the M1 mouse.

We previously reported that repetitive injections of the agonist anti-CD137 mAb in HBV-transgenic mice consistently induced chronic hepatitis, fibrosis, cirrhosis, and, ultimately, adenoma and liver cancer, which closely mimics the pathogenic process of HCC developed from chronic hepatitis[29]. Nine months after five weeks of injection of the anti-CD137 mAb, multiple liver tumor nodules of various sizes were present in all treated mice. The cytological and histological characteristics of both hepatocellular adenoma (HCA) and carcinomas (HCC) were detected. Nodules with normal or larger hepatocytes with little cytoplasm and relatively hyperchromatic nuclei were arranged in one to two cell-thick distorted trabeculae were classified as HCA (Fig 1A), whereas nodules characterized by the uneven proliferation of hepatocytes, hemorrhage and necrosis, were classified as HCC (Fig 1B).

To study the genomic variations underlying primary HCC development, 25 liver tumor nodules larger than 3 mm in diameter and four adjacent non-tumor tissues were harvested by bulk sampling from four mice (M1, M2, M3 and M4) from 10 to 20 months after antibody injections (S1 Table and S1A Fig). The M1 mouse had only 3 macroscopic tumors, whereas the other mice had multiple tumor nodules. Except for six small nodules (T5-T9) from the M3 mouse, all other tissues were histologically analyzed using H&E staining. All analyzed nodules from the M1, M3 and M4 mice were HCC, whereas the six nodules from the M2 mouse included two adenomas (T2 and T5) and four hyperplasias (T1, T3, T 4 and T6) (S2 Fig). We performed WES for three tumor nodules (T) from each mouse, adjacent tissue samples from M2 and M3, and the peripheral blood DNA of M4 because a small tumor was found on M4N after the pathology analysis. M1T1, M1T2 and M1N were also submitted for WGS (S1 Table).

### Genetic diversity among tumors and the independent origin of most tumors in the sequenced mice

For all WES sequenced samples, we obtained 56-fold mean coverage of whole-exome regions, with 84% of loci covered at > 10-fold, whereas the average depth was 23-fold for the whole genome-sequenced samples, with 93% coding regions (CDs) covered at > 10-fold (S2 Table). All candidate somatic SNVs within the CDs were further validated by Sequenom genotyping. In addition to the sequenced samples, 3, 6 and 4 additional tumor nodules were respectively harvested from the M2, M3 and M4 mice and used for validation (S1 Table). Overall, we identified 46 SNVs in the exomes of sequenced tumors, including 32 missense, 12 synonymous, 1 nonsense and 1 splicing mutation. The number of mutations ranged from 0 in nodules from M2T1 and M2T3, which were characterized as hyperplasic, to a maximum of 17 in M1T1. The other 9 tumors had 6 (M4T6), 5 (M1T2), 5 (M2T2), 5 (M4T1), 4 (M3T5), 3 (M1T1), 1 (M1T3), 1 (M3T2), and 1 (M4T4) mutations within CDs (Table 1). Of these mutations, the *Hras* Q61L mutation was the only one shared by three tumors from the M1 mouse. All other SNVs were only verified in the sequenced samples, such that no SNV was recurrent in other tumors. These data indicate the independent origin of the multiple tumors in the mice, except for the M1 mouse.

**Table 1:** Validated somatic SNVs in tumors from mice M1, M2, M3 and M4.

Mouse	Chr.	Position	Mutation	AA change	Genes	Frequency of Sequenom Genotyping										Note
						**T1**	**T2**	**T3**	**N**							
**M1**	**chr7**	**148378449**	**T→A**	**Q->L**	*Hras1*	**0.44**	**0.36**	**0.30**	**0.00**							**T1, T2, T3 common SNV**
	chr1	58349118	T→A	L->H	*Aox3l1*	**0.24**	0.00	0.00	0.00							T1 specific
	chr2	129926854	T→G	L->V	*AU015228*	**0.25**	0.00	0.00	0.00							T1 specific
	chr3	51252263	C→T	A->V	*Naa15*	**0.38**	0.00	0.00	0.00							T1 specific
	chr3	85477153	G→T	R = >R	*Fam160a1*	**0.31**	0.00	0.00	0.00							T1 specific
	chr4	129166348	T→C	E = >E	*Tssk3*	**0.23**	0.00	0.00	0.00							T1 specific
	chr5	100521811	T→G	Q->H	*Tmem150c*	**0.16**	0.00	0.00	0.00							T1 specific
	chr6	43124676	A→C	E->D	*Olfr13*	**0.17**	0.00	0.00	0.00							T1 specific
	chr7	11300559	C→A	P->T	*Nlrp4b*	**0.33**	0.00	0.00	0.00							T1 specific
	chr11	43393263	T→A	F->I	*Ccnjl*	**0.20**	0.00	0.00	0.00							T1 specific
	chr11	98577803	A→C	L->R	*Med24*	**0.18**	0.00	0.00	0.00							T1 specific
	chr12	120688797	G→T	L->R	*Macc1*	**0.15**	0.00	0.00	0.00							T1 specific
	chr13	101399459	T→G	K->T	*Rad17*	**0.26**	0.00	0.00	0.00							T1 specific
	chr14	26691998	G→T	A->S	*Anxa11*	**0.21**	0.00	0.00	0.00							T1 specific
	chr16	38827106	G→A	L = >L	*4930435E12Rik*	**0.17**	0.00	0.00	0.00							T1 specific
	chr17	46284963	A→C	F->V	*Mad2l1bp*	**0.23**	0.00	0.00	0.00							T1 specific
	chrX	56387063	T→G	R = >R	*Fgf13*	**0.40**	0.00	0.00	0.00							T1 specific
	chr1	83273934	C→T	G->S	*Sphkap*	0.00	**0.36**	0.00	0.00							T2 specifc
	chr2	40732203	C→A	SPLICE_ SITE	*Lrp1b*	0.00	**0.38**	0.00	0.00							T2 specifc
	chr4	133975849	G→A	G->D	*Pafah2*	0.00	**0.20**	0.00	0.00							T2 specifc
	chr7	114829397	G→A	V = >V	*Ppfibp2*	0.00	**0.26**	0.00	0.00							T2 specifc
						**T1**	**T2**	**T3**	**T4**	**T5**	**T6**	**N**				T2_specifc
M2	chr3	126141508	G→A	G = >G	*Arsj*	0.00	**0.37**	0.00	0.00	0.00	0.00	0.00				T2_specifc
	chr9	92490151	G→A	D→W	*Plod2*	0.00	**0.26**	0.00	0.00	0.00	0.00	0.00				T2_specifc
	chr13	62976559	G→T	L→I	*Fbp1*	0.00	**0.52**	0.00	0.00	0.00	0.00	0.00				T2 specifc
	chrX	76739033	G→A	R→C	*Gm7173*	0.00	**0.29**	0.00	0.00	0.00	0.00	0.00				T2_specifc
	chrX	86651111	T→A	T = >T	*Pet2*	0.00	**0.35**	0.00	0.00	0.00	0.00	0.00				T2_specifc
M3						**T1**	**T2**	**T3**	**T4**	**T5**	**T6**	**T7**	**T8**	**T9**	**N**	
	chr8	89237354	G→T	K->N	*Lonp2*	**0.24**	0.00	0.00	0.00	0.00	0.00	0.00	0.00	0.00	0.00	T1_specific
	chr16	73948572	C→G	T = >T	*Robo2*	**0.13**	0.00	0.00	0.00	0.00	0.00	0.00	0.00	0.00	0.00	T1_specific
	chr18	61076401	C→T	A->V	*Arsi*	**0.38**	0.00	0.00	0.00	0.00	0.00	0.00	0.00	0.00	0.00	T1_specific
	chr7	149027752	C→A	S->R	*Muc5b*	0.00	**0.17**	0.00	0.00	0.00	0.00	0.00	0.00	0.00	0.00	**T2_specifc**
	chr2	89368270	A→G	L->P	*Olfr1243*	0.00	0.00	0.00	0.00	**0.29**	0.00	0.00	0.00	0.00	0.00	T5_specific
	chr8	46111178	A→C	K->Q	*Fat1*	0.00	0.00	0.00	0.00	**0.09**	0.00	0.00	0.00	0.00	0.00	T5_specific
	chr11	93954940	A→T	N->I	*Spag9*	0.00	0.00	0.00	0.00	**0.08**	0.00	0.00	0.00	0.00	0.00	T5_specific
	chr16	44729724	G→T	R->L	*BC027231*	0.00	0.00	0.00	0.00	**0.22**	0.00	0.00	0.00	0.00	0.00	T5_specific
M4						**T1**	**T2**	**T3**	**T4**	**T5**	**T6**	**T7**	**Blood**			
	chr2	23942697	T→C	K = >K	*Tbpl2*	**0.18**	0.00	0.00	0.00	0.00	0.00	0.00	0.00			T1_specific
	chr2	85420073	T→G	L->V	*Olfr996*	**0.15**	0.00	0.00	0.00	0.00	0.00	0.00	0.00			T1_specific
	chr8	107881502	G→A	A->T	*Slc9a5*	**0.09**	0.00	0.00	0.00	0.00	0.00	0.00	0.00			T1_specific
	chr8	112478417	T→C	M->V	*Marveld3*	**0.16**	0.00	0.00	0.00	0.00	0.00	0.00	0.00			T1_specific
	chr14	70656177	G→A	F = >F	*Ppp3cc*	**0.44**	0.00	0.00	0.00	0.00	0.00	0.00	0.00			T1_specific
	chr2	60281361	G→A	R->*	*Pla2r1*	0.00	0.00	0.00	**0.19**	0.00	0.00	0.00	0.00			T4_specific
	chr7	29860191	A->G	F->S	*Ryr1*	0.00	0.00	0.00	0.00	0.00	**0.13**	0.00	0.00			T6_specific
	chr7	108652898	G->T	S = >S	*Pde2a*	0.00	0.00	0.00	0.00	0.00	**0.23**	0.00	0.00			T6_specific
	chr8	18934246	C→T	D->N	*Xkr5*	0.00	0.00	0.00	0.00	0.00	**0.17**	0.00	0.00			T6_specific
	chr10	80643593	A->G	E = >E	*Eef2*	0.00	0.00	0.00	0.00	0.00	**0.15**	0.00	0.00			T6_specific
	chr13	103525596	G→A	S->L	*Mast4*	0.00	0.00	0.00	0.00	0.00	**0.42**	0.00	0.00			T6_specific
	chr19	45228206	A->T	R->W	*Tlx1*	0.00	0.00	0.00	0.00	0.00	**0.21**	0.00	0.00			T6 specific

The three tumors of the M1 mouse shared the *Hras* mutation, suggesting that they have a common origin. Their phylogenetic relationship was constructed based on the validated point mutations (Fig 1C). In addition to the *Hras* mutation, T1 and T2 had 16 and 4 of their own SNVs, respectively, whereas no extra SNV was identified in T3. To verify the SNV distribution within tumors, we validated the SNVs of T1 and T2 in 12 and 19 micro-dissected samples of the T1 and T2 tumors, respectively. Most of the SNVs were validated by Sequenom genotyping, except a few that failed (S4 Table). For SNV frequencies that were validated in micro-dissected samples, there was no significant difference among micro-dissected samples and the bulk sample in T1 (Kruskal-Wallis test, *P value*, 0.99) and T2 (Kruskal-Wallis test, *P value*, 0.81). The specific SNVs for each tumor were nearly validated in all micro-sections of the tumor, suggesting that these specific mutations accumulated at a very early stage of clone expansion.

For the whole-genome level variations of tumors from M1, we identified 1163 and 943 SNVs for M1T1 and M1T2, respectively. Only 56 SNVs, representing 6% of the total SNVs in M1T1 and M1T2, were shared by both tumors (S3 Table). For the liver specimen, M1T1 and M1T2 were located on the middle liver lobe, whereas T3 was found on a margin of the left liver lobe (Fig 1D). The common origin of the three tumors from M1 suggests that their ancestor cell population was able to migrate within the liver at a very early stage prior to tumor cell population expansion. To define the chromosomal aberrations in the tumorigenesis of primary HCC, we assessed somatic CNAs of M1T1 and M1T2 based on the read depth of the whole genome sequencing data compared to that of the normal control M1N. A large fraction of the genome had undergone alterations in M1T1, including deletions of Chr4, Chr8, Chr9, Chr13, Chr14, and Chr17 and a 45 M region within ChrX and gains of Chr7, a 50 M region of Chr10, Chr15, and Chr16 and a region of approximately 50 M of Chr18 (S3 Fig). However, no obvious CNA was found in M1T2. SVs of M1T1 and M1T2 were predicted based on the whole genome sequencing data. After validation of the deletions by PCR and Sanger sequencing, we found one 57 kb deletion, including the second exon of *Gpr19* and exons 1–3 of *Cdkn1b* on Chr6; two deletions of 257 kb, including exons 28–37 of *Gtf3c1* and exons 1–28 of *D430042o09rik*, and of 25 kb containing exons 1–11 of *Gsk3a* and exons 1–4 of *Erf* on Chr7; and one deletion approximately 57 kb, comprising exons 2–5 of *Egfr* on Chr11 in M1T1 (S5 Table). No SVs were found in M1T2. The more frequent occurrence of CNAs and SVs in M1T1 is consistent with it having the most SNVs, suggesting that M1T1 is more genomically unstable than M1T2.

### Allele fractions of SNVs, tumor cell fraction and cell ploidy in liver tumors with multiple mutations

**Fig 2:**
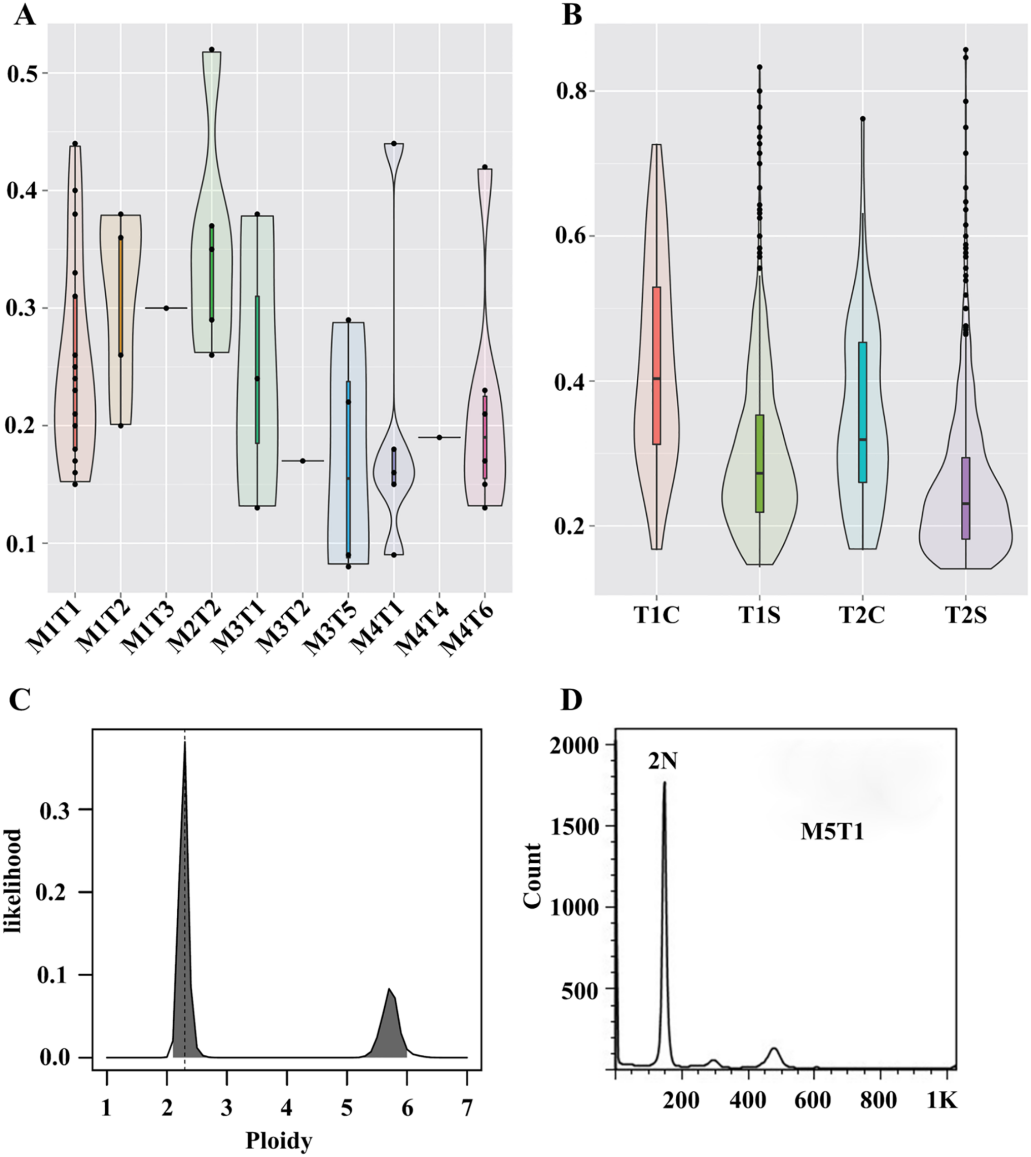
Allelic fraction density and ploidy of tumor cells. Violin plots illustrating the allelic fraction density of validated somatic mutations in each tumor (A) and of the estimated SNVs at the whole genome level in M1 (B). (C) Estimated ploidy of M1T1 based on sequencing data by Sequenza. (D) DNA ploidy of M5T1, as determined by flow cytometry.

In tumors, somatic mutations of similar frequencies may reside in the same population of cells, which may have descended from the same founder; therefore, the clustering of mutation frequency may represent different subclones within a single tumor[17]. Although we only detected 46 SNVs, we found that the allele fraction of different SNVs varied from low (approximately 0.1) to high (> 0.3) in the same tumors that contained more than 3 mutations in the CDs, such as M1T1, M1T2, M2T2, M3T1, M3T5, M4T1 and M4T6 (Table 1). We used violin plots to illustrate the allelic fraction densities of somatic mutations in each tumor. The violin plots of SNV distributions indicate the existence of subclones in those tumors with multiple SNVs (Fig 2A).

For the whole-genome level variations of M1T1 and M1T2, the 56 SNVs shared by both tumors had a higher frequency than their specific SNVs. Using allele fractions of 0.3 as the cut-off point, we found that 82% of shared mutations in T1 were above the cut-off, with a mean value of 0.42, and 59% of common mutations in T2 were above the cut-off, with a mean value of 0.35 (S4 Table and Fig 2B). The relatively higher allele fraction, 0.35 to 0.42, of their shared mutations suggests that the tumor cell fraction of those two samples is quite high, approximately 70% to 82%, which is consistent with tumor cell purities of 70%-90% in clinical HCC samples[9]. The allele fraction of specific SNVs below the 0.3 cut-off was 60% in M1T1 and 75% in M1T2. Moreover, the average allele fraction was 0.23 for M1T1 and 0.25 for M1T2 (S4 Table and Fig 2B). The allele fraction was relatively higher for shared SNVs and lower for specific SNVs in M1, suggesting that the specific mutations were gained after splitting from their common ancestor and indicate the existence of subclones in M1T1 and M1T2 (Fig 2B).

However, when we used the sequence data without distinguishing between common mutations and specific mutations in M1T1 and M1T2, the tumor cell purity was estimated to be approximately 50% because most SNVs have a medium allele fraction of 0.2–0.25, which is consistent with results estimated using only tumor-specific mutations. In addition, except for M4T1 and M4T4, which were polyploid, the karyotypes of all other tumors were nearly diploid, as estimated based on the sequenced dataset (Fig 2C and S4A Fig), although abundant CNAs were found in M1T1. The estimated diploid karyotypes were confirmed by flow cytometry analysis with a diploid lymphoma cell line as a control (Fig 2D and S4B Fig).

### Functional annotation of the mutated genes

**Fig 3:**
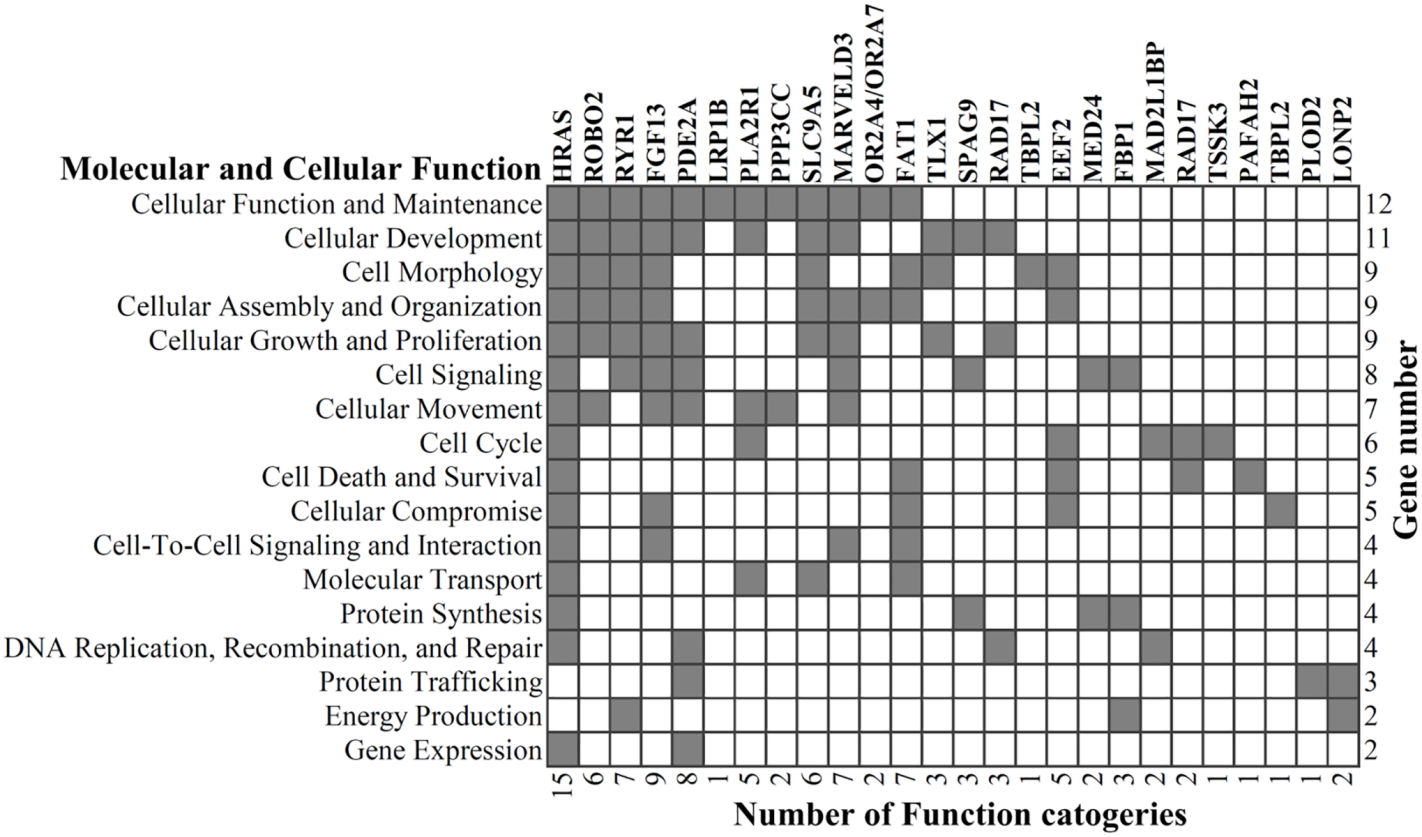
Cellular and molecular functional analysis of the mutated genes. Ingenuity Pathway Analysis (IPA) was used to investigate the molecular/cellular functions of the 46 mutated genes, 26 of which were mapped to 17 terms that describe different molecular and cellular functions.

Most individual liver tumors only have 1–6 mutated genes that have been identified in liver tumor models, and the question is whether these mutated genes are drivers for tumorigenesis. To evaluate their tumorigenic capacity, we used Ingenuity Pathway Analysis (IPA) to investigate molecular/cellular functions, diseases and disorders based on all 46 mutated genes. A total of 26 mutated genes were mapped to 17 terms that describe different molecular and cellular functions, such as cellular function and maintenance, cellular development, cell morphology, cellular assembly and organization, cellular growth and proliferation, cell signaling, cellular movement, cell cycle, cell death and survival (Fig 3). We also found 37 genes related to different types of cancer in the IPA database, and the functional annotation showed that they are associated with cancer-specific biological processes, such as tumorigenesis, transformation, development, and invasion (S6 Table). In summary, most mutated genes found in the mouse HCC samples may play important roles in tumor progression or suppression, although the mechanism by which these mutations contributes to the tumorigenesis of HCC requires further studies.

### Mutation frequency and expression of mutated genes in clinical HCC samples

**Fig 4:**
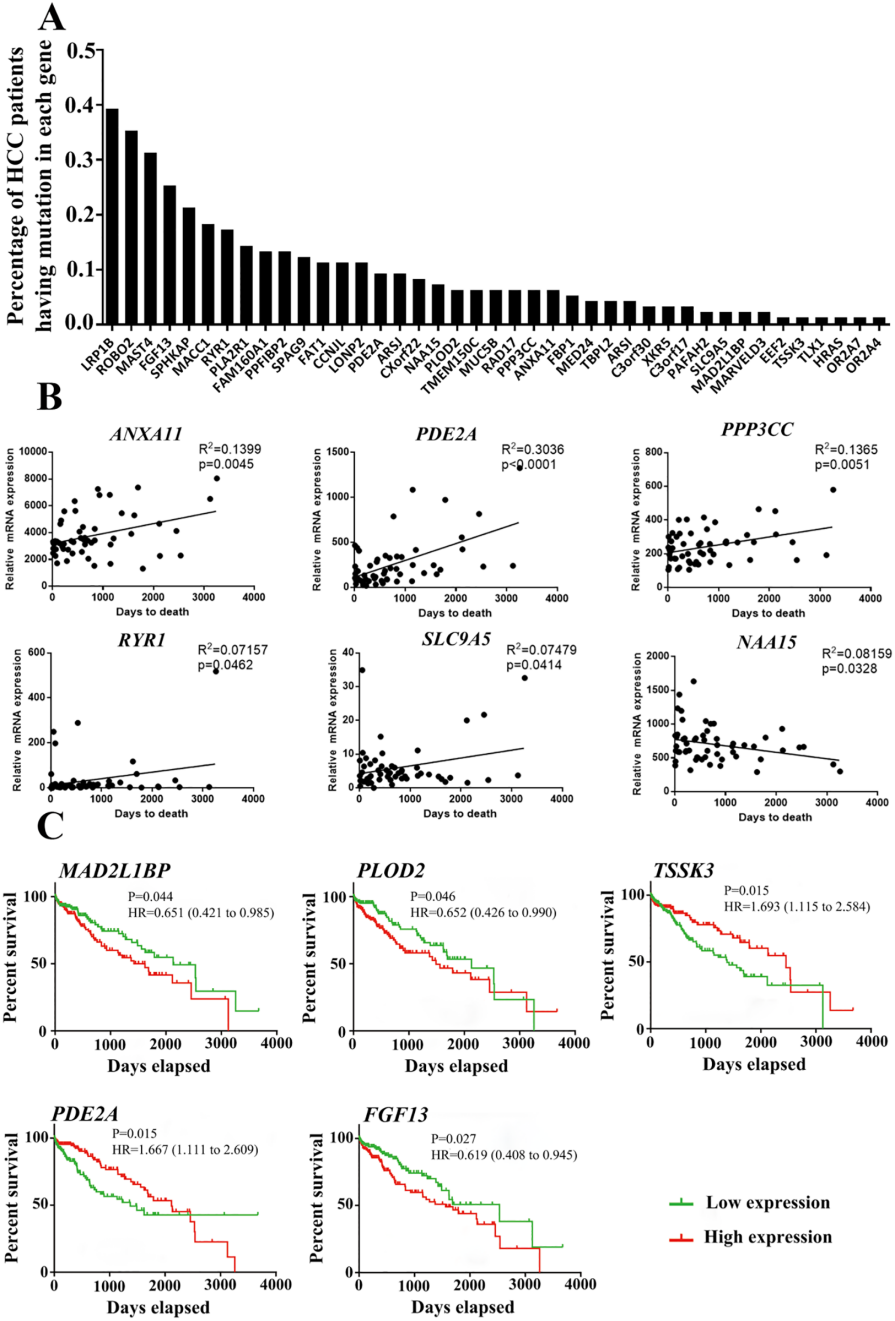
Mutation frequency, gene expression and clinical outcome analysis based on the ICGC database. (A) Mutation percentage of the mutated genes found in the mouse model and patients with liver tumors. (B) Correlation of the gene expression level of *ANXA11*, *PDE2A*, *PPP3CC*, *RYR1*, *SLC9A5*, and *NAA15* and overall survival for patients with liver cancer. *P* values are based on Spearman’s rank test (two-sided). The solid lines represent a linear trend. (C) Kaplan–Meier survival curves based on *TSSK3*, *PDE2A*, *MAD2L1BP*, *PLOD2* and *FGF13* expression. The median gene expression value was used as a cut-off point for each gene to divide patients into high and low gene expression groups. The hazard ratio with 95% confidence interval and *P* value of the log-rank test are given for each gene.

The number of mutated genes found in mouse HCC samples was lower than in clinical HCC samples. To explore the mutation frequency in human HCCs, 1128 patients with liver tumors from six research projects of the ICGC were enrolled (S7 Table). Of the 46 mutated genes in the mouse liver tumor, all 41 human homolog genes were mutated in at least one patient (Fig 4A). *LRP1B* was the most frequently mutated gene (359 of 926 patients), followed by *ROBO2*, *FGF13*, *MAST4* and *SPHKAP*, which were mutated in more than 20% of patients (Fig 4A and S8 Table). *LRP1B* is a tumor suppressor and may regulate cell motility via the *RhoA*/*Cdc42* pathway and actin cytoskeleton reorganization[30], but the exact role of *LRP1B* in HCC development has not been reported. *ROBO2* is also a candidate tumor suppressor gene[31]; thus far, there is no clear evidence that *FGF13*, *MAST4* and *SPHKAP* are associated with cancer development.

For the gene expression and clinical outcome analysis, we used Spearman’s rank test (*P* < 0.05) to analyze the correlation between global gene expression and patient survival. The results showed that the expression of *ANXA11*, *PDE2A*, *PPP3CC*, *RYR1* and *SLC9A5* was positively correlated with the patient survival, whereas expression of *NAA15* was negatively correlated with patient survival (Fig 4B). To further elucidate the relationships between the differential expression of these genes and patient survival, two patient groups with high and low expression of each gene were compared using a Kaplan-Meier survival plot. Based on log-rank *P* values < 0.05, patients with high expression of *TSSK3* or *PDE2A* had a longer survival time than those with low expression of both genes; in contrast, patients with high expression of *MAD2L1BP*, *PLOD2* or *FGF13* tended to have a shorter survival time than those with low expression of these genes (Fig 4C). Of these genes, *PLOD2* expression has been significantly correlated with tumor size and macroscopic intrahepatic metastasis and has also been identified as a significant, independent factor of poor prognosis[32].

## Discussion

With advances in next generation sequencing technologies, an increasing number of studies have demonstrated the extensive genetic variations of HCC[2–7,11,33]. Because most studies to date were conducted on surgically resected tumors, we have little knowledge of the genetic alterations that occur in early lesions. Sequencing spontaneous tumors during an early stage from a mouse model will improve our understanding of the genes and pathways that are involved in the etiology of HCC. In this study, we performed WGS and WES to identify genetic variations in spontaneous early-stage HCCs that arise in the context of chronic hepatitis in inbred mice. We sequenced 12 liver tumors from 4 mice and detected 46 SNVs. Except for the *Hras* mutation, which was shared by three tumors from M1, no SNVs were recurrent in other tumors, even those obtained from the same mouse for validation, indicating the independent origin of tumors and the high heterogeneity of inter-tumors in this hepatitis-related primary HCC mouse model.

The primary goal of cancer genomic sequencing is to identify cancer driver genes that lead to tumor development. A common method for identifying driver mutations is to find recurrent mutations or currently mutated genes with significant frequency in a large cohort of human cancer samples. Because this method requires a large enough cohort of samples and because many driver genes are mutated at a low frequency, it is difficult or impossible to distinguish driver mutations from passenger mutations, which are functionally neutral and do not contribute to tumorigenesis, based on frequency alone. Compared with the average number of approximately 50 protein-changing mutations per individual tumor based on clinical samples[3–6], the mutation number was lower in this mouse HCC model. Except for in the M1T1 tumor, we only detected 1 to 6 mutations within the CDs in individual tumors. Although some low-frequency mutations might not be detected because our sequencing depth was not high, these results demonstrated an extreme background of “passage mutation” in the inbred mouse tumor model, which is an advantage for the identification of cancer driver genes.

Among the 46 identified mutated mouse genes, 41 homologous genes were found in the human genome database, and 37 genes were associated with cancer-specific biological processes in different types of cancer based on the IPA database. All 41 human homolog genes were mutated in patient HCCs from six research projects of the ICGC. *LRP1B* was mutated in nearly 40% of patients with HCC, followed by *ROBO2*, *FGF13*, *MAST4* and *SPHKAP*, which were mutated in more than 20% of patients. *LRP1B* and *ROBO2* are tumor suppressors[30,31], but FGF13, *MAST4* and *SPHKAP* have not been associated with cancer development. In addition, *Hras* is a well-known proto-oncogene implicated in a variety of cancers[34]. Its Q61L mutation was identified in three tumors from the M1 mouse, and this mutation may have a global impact on the structures of both Ras and Raf-RBD in the complex, which can contribute to oncogenesis beyond local effects on the active site[35]. These results suggest that these mutated genes are potentially involved in tumorigenesis of the primary mouse HCC, although their roles in tumor development have yet to be studied individually.

In addition to genetic changes, epigenetic abnormalities can also result in dysregulated gene expression and function[36]. Epigenetic changes, such as global DNA hypomethylation and specific promoter hypermethylation, have been linked with genomic instability and inactivation of tumor suppressor genes, respectively[36,37], and both are commonly observed in benign neoplasia nodules and early-stage tumors[36,37]. In the mouse model, we only detected 1 to 6 mutations at the CDs in individual tumors, and none were recurrent, which is similar to the mutation pattern of a few recurrently mutated genes found in childhood tumors, such as medulloblastoma[38], neuroblastoma[39] and rhabdoid tumours[40]. Parker *et*. *al*.[41] and Mack *et*. *al*. [42]found that one type of ependymoma brain tumor lacks tumor-driving mutations but also has aberrant epigenetic modifications, and another type shows neither gene mutations nor epigenetic aberrations. These results suggest that epigenetic alterations could be a preliminary step in tumorigenesis, but it will be challenging to test the mechanisms by which epigenetic modifications drive tumor development. The chronic hepatitis murine model, which mimics the pathogenic process of HCC that develops from chronic hepatitis, could serve as a good model for deciphering the epigenetic changes of early-stage tumors, which may provide new insights into the dynamics of early-stage tumor evolution.

## Materials and methods

### Primary HCC mouse model and collection of the HCC tissues

HBV-transgenic mice C57BL/6J-TgN (Alb1 HBV) 44Bri [43] were purchased from the Jackson Laboratory (Bar Harbor, ME) and maintained under specific pathogen-free conditions in the animal facility at the Institute of Biophysics, Chinese Academy of Sciences. HBV-transgenic mice, 2 months old, were intraperitoneally injected with 100 mg of anti-CD137 Ab (clone 2A) weekly up to five times[29]. Four male mice (M1, M2, M3 and M4) were euthanized at aged 13 months or older, and all nodules on the liver larger than 3 mm in diameter were subjected to bulk sampling (S1A Fig). In addition to one sample obtained from each tumor, 15 and 22 micro-sections (each section is approximately 20,000 cells) were obtained from two tumors (M1T1 and M1T2) in the M1 mouse (S1B Fig) by performing micro-dissections[11]. All mouse and tumor sample information is listed in S1 Table. All studies involving animals were approved by the Institutional Laboratory Animal Care and Use Committee at the Institute of Biophysics, Chinese Academy of Sciences.

### Pathology analysis

Pathological analysis was performed in all tissues used for sequencing to confirm the occurrence of tumors. After paraffin embedding, tissue sections (5 μm) were stained with hematoxylin and eosin (H&E).

### Library preparation, whole-exome capture, WGS and WES

Genomic DNA from bulk samples and micro-sections was extracted using the QIAamp DNA Mini Kit (Qiagen, Hilden, Germany) and TIANampMicro DNA Kit (Tiangen, Beijing, China), respectively. Libraries for the samples from the M2, M3 and M4 mice were constructed using the traditional method with 3 μg DNA as each input, which was sheared to generate fragments between 200 and 300 bp. DNA fragments were end-repaired, ligated with adapters, and amplified following the standard protocol of Paired-End DNA Sample Prep Kit (Illumina). To prepare the libraries from the M1 mouse samples, we used the modified EZ-Tn5 transposase-based method to fragment double-stranded DNA, with 20 ng genomic DNA as each input[11]. After fragmentation, we amplified the libraries used for exome capture and the WGS of M1 with 8 and 10 cycles, respectively. The amplified libraries were purified using the QIAquick Gel Extraction Kit (Qiagen).

Four DNA libraries from each mouse were barcoded with different indexes and equally pooled together. According to the manufacturer’s instructions, 800 ng of pooled DNA libraries were captured using the SureSelectXT Mouse All Exon Kit (Agilent), except custom blockers were used for the M1 libraries[11]. The captured libraries were amplified by PCR for 10 cycles and purified using the QIAquick Gel Extraction Kit (Qiagen). The insert size and the concentration of purified libraries for sequencing were examined using an Agilent Bioanalyzer and qRT-PCR. Paired-end (2×100 bp) multiplex sequencing of samples on the Illumina HiSeq2000 platform was performed.

### Detection of somatic SNVs

Paired-end reads in FastQ format were aligned to the mouse reference sequence (mm9) using the Burrows-Wheeler Aligner (BWA)[44]. The Genome Analysis Toolkit (GATK) was used to re-calibrate the read quality[45], and Picard was used to mark the reads from PCR duplicates. WGS and WES data statistics are given in S2 Table.

With the normal control, somatic SNVs for each tumor were detected using Samtools[46] and VarScan[47]. WGS data from M1N served as a normal control to call SNVs from M1 WES data. In addition to Varscan’s built-in filters, the following filtering criteria were applied to identify candidate somatic mutations of WES: (i) a minimum of 10× coverage required in both tumor and normal samples, (ii) variant present on both strands with total reads ≥ 3 in the tumor, (iii) a variant allele frequency (VAF) in tumor DNA ≥ 10%, (iv) reads with more than two variants were removed, and (vii) variants listed in dbSNP132 were removed. For the WGS data for M1, we used the following criteria to filter SNVs: (i) a minimum of 10× coverage required in both tumor and normal sample, (ii) variant present on both strands with total read ≥ 4 in the tumor, and (iii) a VAF in the tumor ≥ 14%. In addition, we manually checked all candidate SNVs at CDs, which were submitted for Sequenom genotyping validation. All validated SNVs are shown in Table 1, and the SNVs of M1T1 and M1T2 at the whole genome level are presented in S3 Table.

### SNVs validation by Sequenom genotyping

Genomic positions for all validated SNVs were retrieved using mm9 as a reference. The detailed procedures of primer design, multiplexed PCR and allele-specific extension, and VAF calculation of Sequenom genotyping were performed according to Ling *et al*.[11]. After validation, we used the R package ggplot to draw the violin plots to illustrate the allelic fraction densities of somatic mutations in each tumor, i.e., the width of the shaded area represents the proportion of data located there. For the SNVs validated in micro-dissected samples, we used the Kruskal-Wallis test[48] to compare their frequencies among all micro-dissected samples and the bulk sample in T1 and T2.

### Detection of CNAs and SVs and estimation of tumor cell purity and ploidy

Sequenza was used to detect the somatic CNAs and to estimate tumor cell purity and ploidy[49]. First, we used Samtools to convert the Bam file of DNA sequencing data into the Pileup format. Second, the paired tumor and normal Pileup files were processed by sequenza-utils, which extracts sequencing depth, determines homozygous and heterozygous positions in the normal specimen, and calculates the variant alleles and allelic frequency from the tumor specimen. The sequenza-utils output was further processed using the Sequenza R package 2.1.1 to provide the segmented copy number data, cellularity, and ploidy estimates for each sample. We used Crest[50] to detect the SVs in M1T1 and M1T2 based on the WGS data. Deletions were further validated by PCR and Sanger sequencing.

### DNA ploidy analysis by flow cytometry

Two tumors and a non-tumor tissue sample from the M5 mouse were mechanically dissociated in phosphate-buffered saline followed by filtration through a piece of fine nylon mesh (75 μm pore size) and centrifugation to remove debris and cell clumps. The single cell suspensions were fixed in cold 70% ethanol followed by staining using propidium iodide (Sigma) (50 g/ml in PBS) as a DNA-specific fluorochrome. Flow cytometric analysis was performed with BD FACSCalibur.

### Functional analysis of the mutated genes

IPA was used to analyze the 46 mutated genes for their molecular/cellular functions and relationship with diseases and disorders. To explore the clinical significance of the mutated genes, we used ICGC data to investigate the mutation rates of these genes in human HCC. A total of 1128 patients with HCC from six projects (Liver Hepatocellular carcinoma—TCGA, Liver Cancer—FR, Liver Cancer—RIKEN, Liver Cancer—NCC, Benign Liver Tumour, and Liver Cancer—Hepatocellular macronodules) were included.

### Statistical analysis

Statistical analysis was performed using GraphPad Prism 6.0 (GraphPad Software, Inc). Spearman’s rank test (two-sided) was used to analyze the correlation of the gene expression level and overall survival for patients with liver cancer. In addition, we used the median gene expression value as the bifurcating point for each gene to divide patients into high and low gene expression groups. The two patient groups were compared using a Kaplan-Meier survival plot for each gene, and the hazard ratio with 95% confidence intervals and log-rank *P* value were calculated.

## Supporting information

S1 Checklist ARRIVE guidelines checklist. (DOCX)

S1 Fig Tumor samples found in a chronic hepatitis murine model. (A) Liver tumor nodules harvested by bulk sampling from M1, M2, M3 and M4 and (B) sample collection with micro-dissection performed in M1T1 and M1T2.
(JPG)

S2 Fig Pathology analysis of liver tumor sections stained with H&E. Yellow arrows indicate uneven proliferation of hepatocytes, and green arrows indicate enlarged hepatocytes.
(JPG)

S3 Fig Copy number variations of M1T1. CNVs were called with Sequenza, which is based on the read depth of the whole genome sequencing data compared with the normal control.
(JPG)

S4 Fig Tumor cell karyotypes. (A) Estimated karyotype of tumor cells based on sequencing data, and (B) karyotype of tumor cells determined by flow cytometry.
(JPG)

S1 Table Tumor samples from the HBV transgenic mice treated with the anti-CD137 Ab. (XLSX)

S2 Table Whole genome sequencing (WGS) and whole exome sequencing (WES) data statistics. (XLSX)

S3 Table Validation results of somatic SNVs in the micro-section samples from M1T1 and M1T2. (XLSX)

S4 Table Somatic SNVs of M1T1 and M1T2 at the whole genome level. (XLSX)

S5 Table Validated structure variations in M1T1. (XLSX)

S6 Table Genes associated with cancer-specific biological processes from the IPA database. (XLSX)

S7 Table Summary of the 1128 patients with liver tumors from the ICGC who were used to verify the mutation frequency and expression of these mutated genes in clinical HCC. (XLSX)

S8 Table Mutation frequency of the mutated genes found in the HBV transgenic mouse model and in 960 patients with liver tumors from the ICGC. (XLSX)
