# Supplementary material for: Genomic sequencing identifies a few mutations driving the independent origin of primary liver tumors in a chronic hepatitis murine model
Source: PLoS One. 2017 Nov 8;12(11):e0187551. doi: 10.1371/journal.pone.0187551 (PMC5678715; doi:10.1371/journal.pone.0187551)

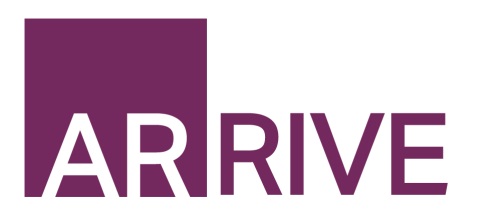


The ARRIVE Guidelines Checklist

Animal Research: Reporting In Vivo Experiments

Carol Kilkenny^1^, William J Browne^2^, Innes C Cuthill^3^, Michael Emerson^4^ and Douglas G Altman^5^

*^1^The National Centre for the Replacement, Refinement and Reduction of Animals in Research, London, UK, ^2^School of Veterinary Science, University of Bristol, Bristol, UK, ^3^School of Biological Sciences, University of Bristol, Bristol, UK, ^4^National Heart and Lung Institute, Imperial College London, UK, ^5^Centre for Statistics in Medicine, University of Oxford, Oxford, UK.*

|  | ITEM | RECOMMENDATION | | Section/ Paragraph | |
| --- | --- | --- | --- | --- | --- |
| 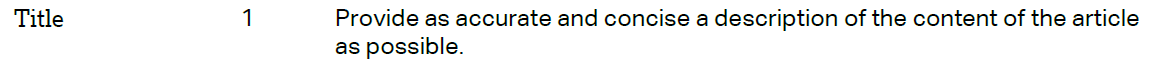 | | | Title | |  |
| 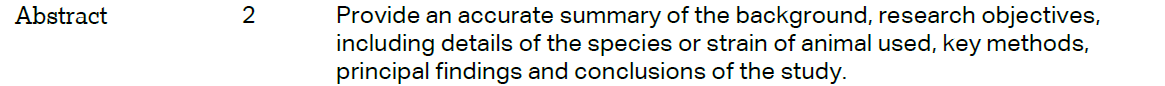 | | | Abstract | |  |
| INTRODUCTION | | |  | |  |
| 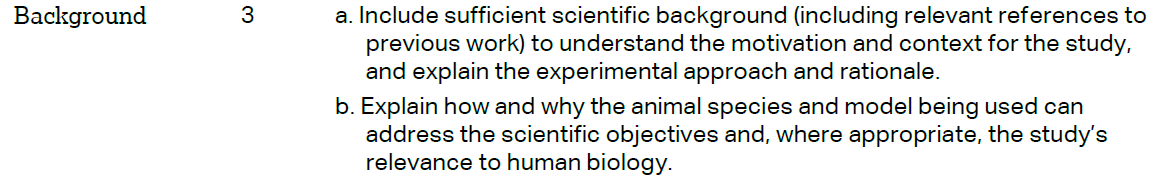 | | | Introduction:  Paragraphs 1-3 | |  |
| 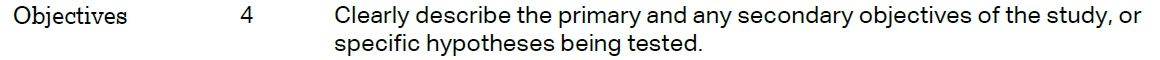 | | | Introduction:  Paragraph 3 | |  |
| METHODS | | |  | |  |
| 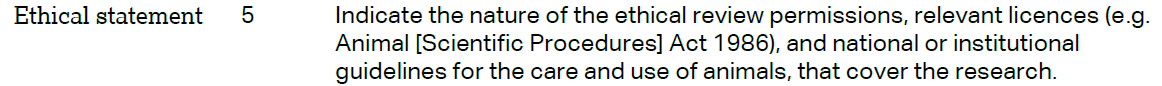 | | | Materials and methods:  Paragraph 1 | |  |
| 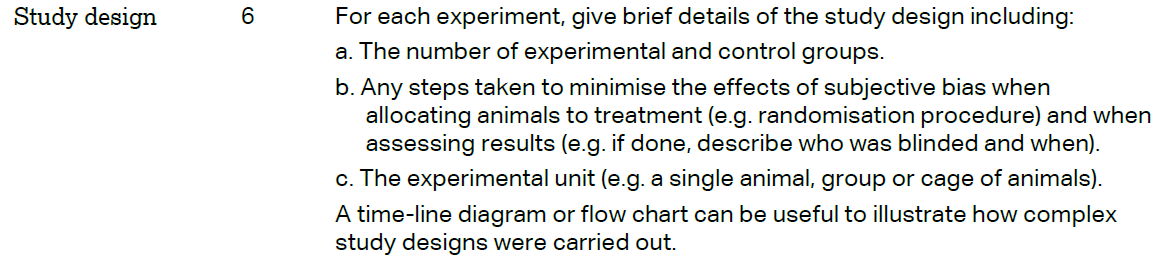 | | | Materials and methods:  Paragraph 1 | |  |
| 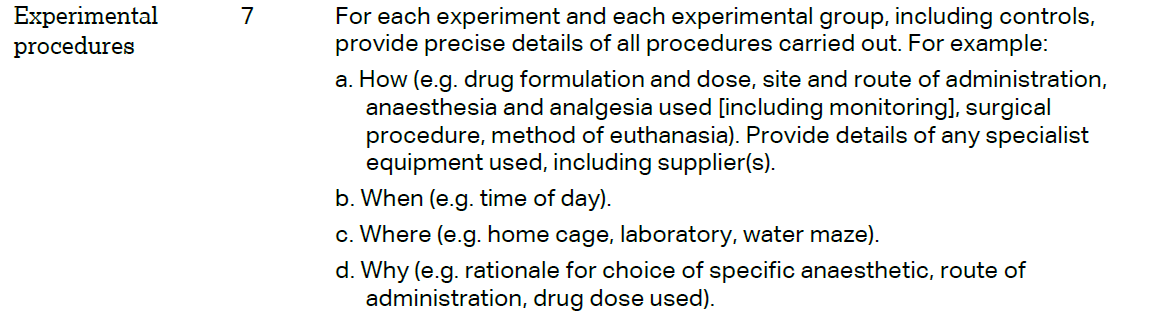 | | | Materials and methods:  Paragraph 1 | |  |
| 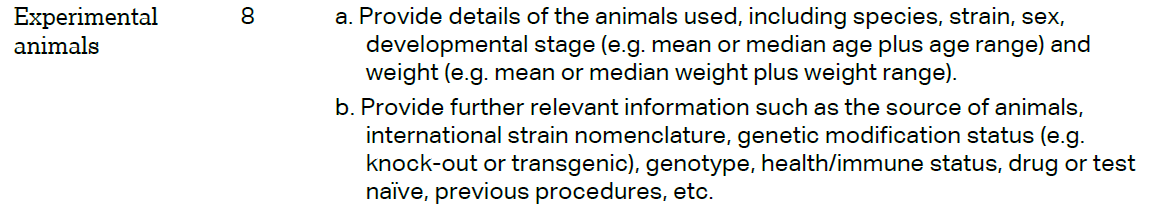 | | | Materials and methods:  Paragraph 1 | |  |

The ARRIVE guidelines. Originally published in *PLoS Biology*, June 2010^1^

| 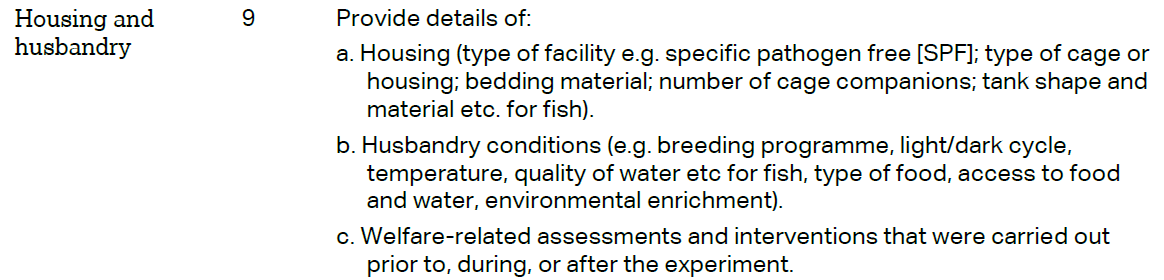 | Materials and methods:  Paragraph 1 | |
| --- | --- | --- |
| 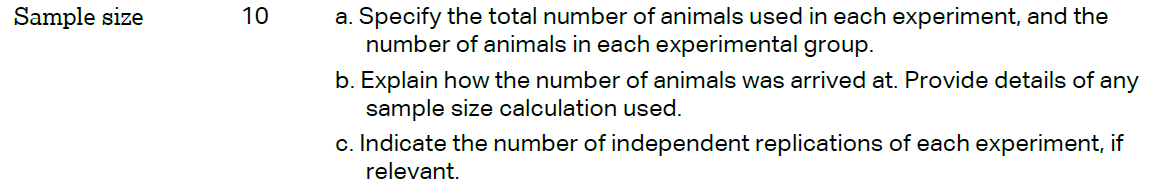 | Materials and methods:  Paragraph 1 | |
| 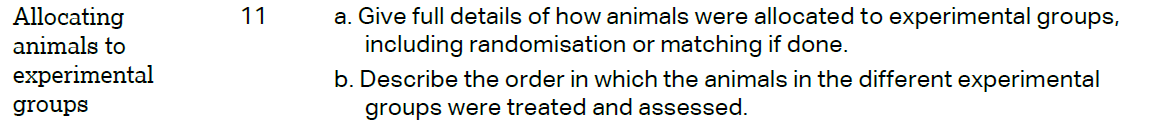 | Materials and methods:  Paragraph 1 | |
| 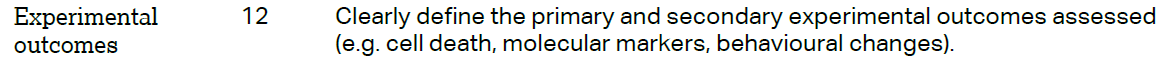 | Materials and methods:  Paragraphs 2-7 | |
| 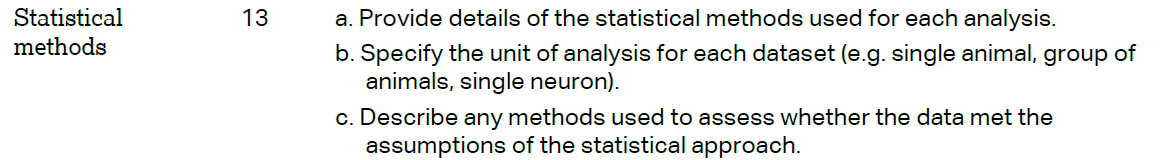 | Materials and methods:  Paragraph 9 | |
| RESULTS |  | |
| 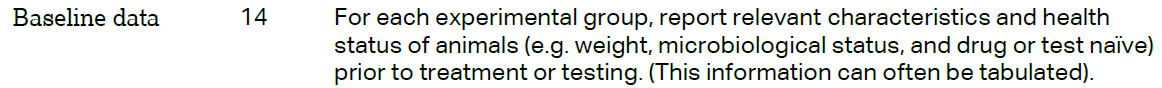 |  | |
| 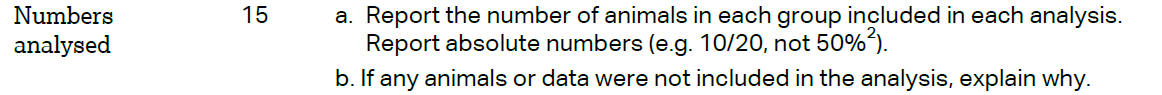 | Results:  Section 1 | |
| 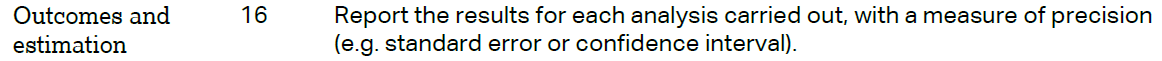 | Results:  Sections 1-3 | |
| 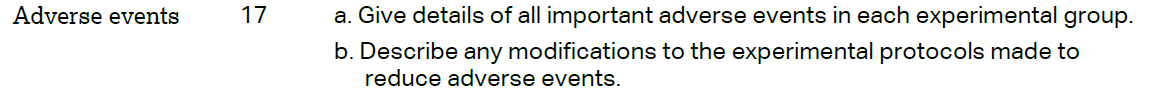 |  | |
| DISCUSSION |  | |
| 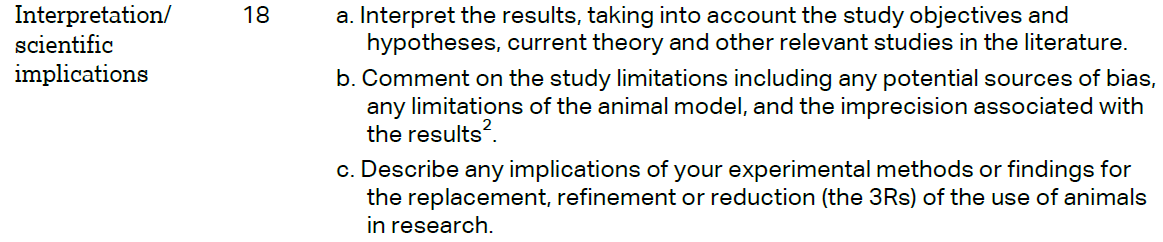 | Throughout | |
| 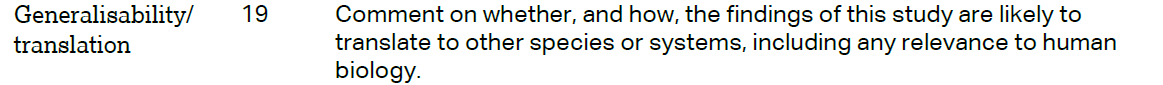 | Discussion  Paragraphs 1,3&4 | |
| 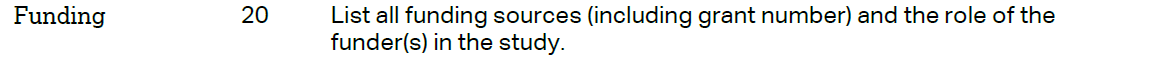 | | Section of Acknowledgments |


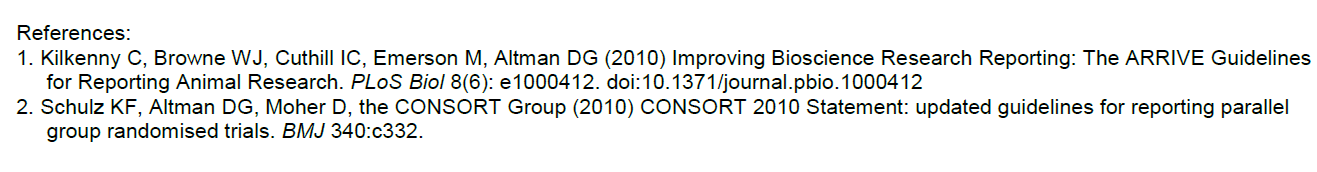

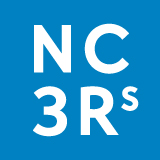

Supplement: S1 Checklist — (DOCX) [file pone.0187551.s001.docx]
