# Supplementary figures and images for: Genomic sequencing identifies a few mutations driving the independent origin of primary liver tumors in a chronic hepatitis murine model
Source: PLoS One. 2017 Nov 8;12(11):e0187551. doi: 10.1371/journal.pone.0187551 (PMC5678715; doi:10.1371/journal.pone.0187551)

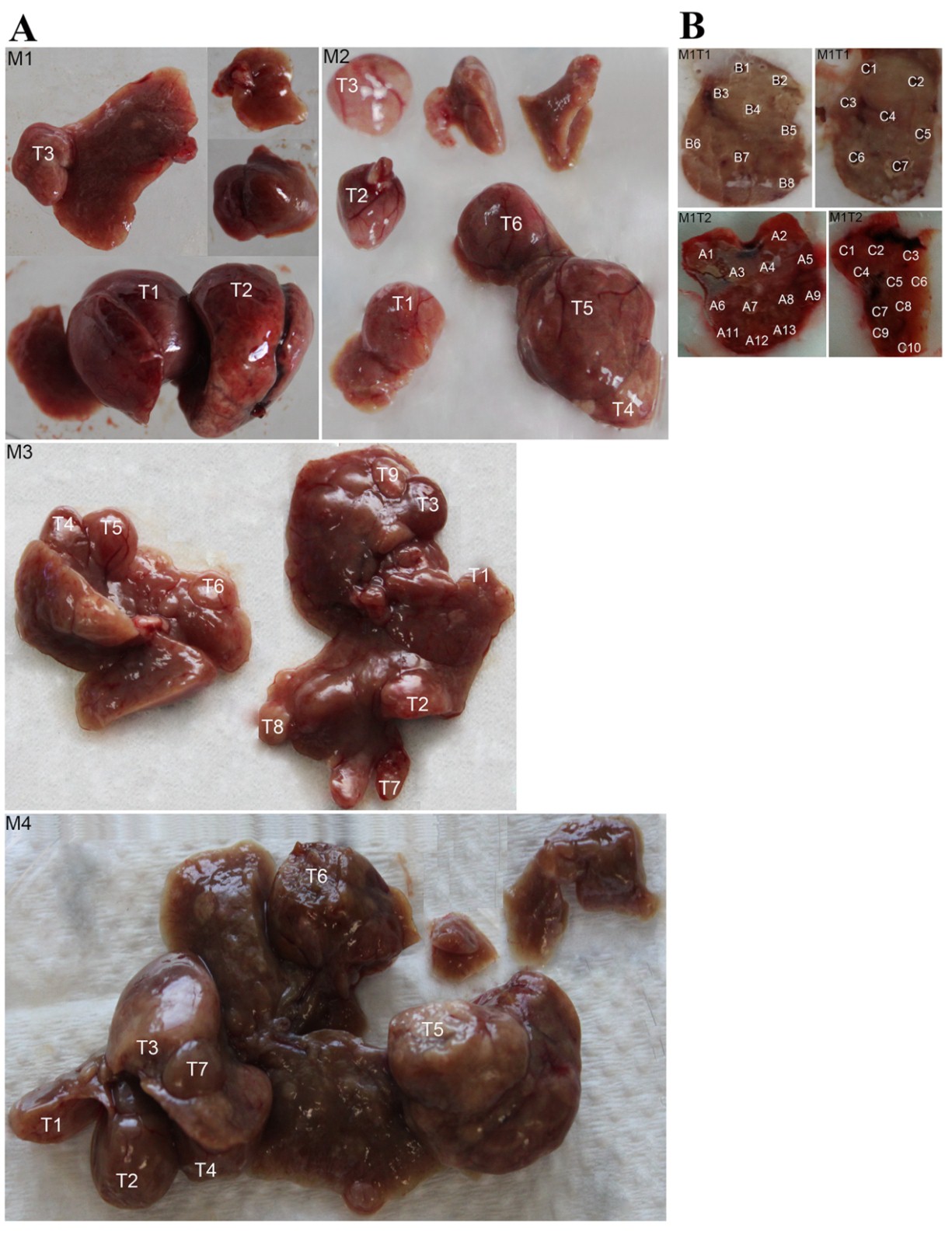

Supplement: S1 Fig — (A) Liver tumor nodules harvested by bulk sampling from M1, M2, M3 and M4 and (B) sample collection with micro-dissection performed in M1T1 and M1T2. (JPG) [file pone.0187551.s002.jpg]

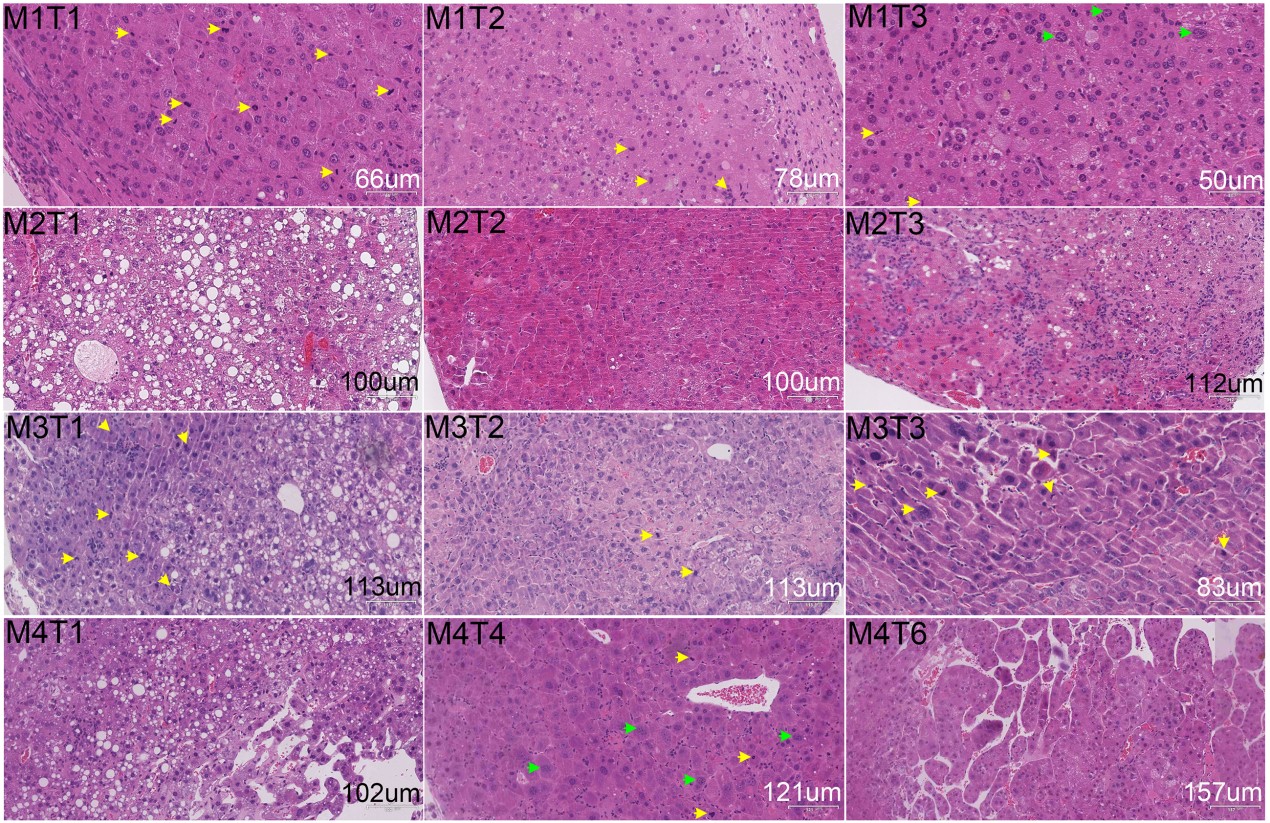

Supplement: S2 Fig — Yellow arrows indicate uneven proliferation of hepatocytes, and green arrows indicate enlarged hepatocytes. (JPG) [file pone.0187551.s003.jpg]

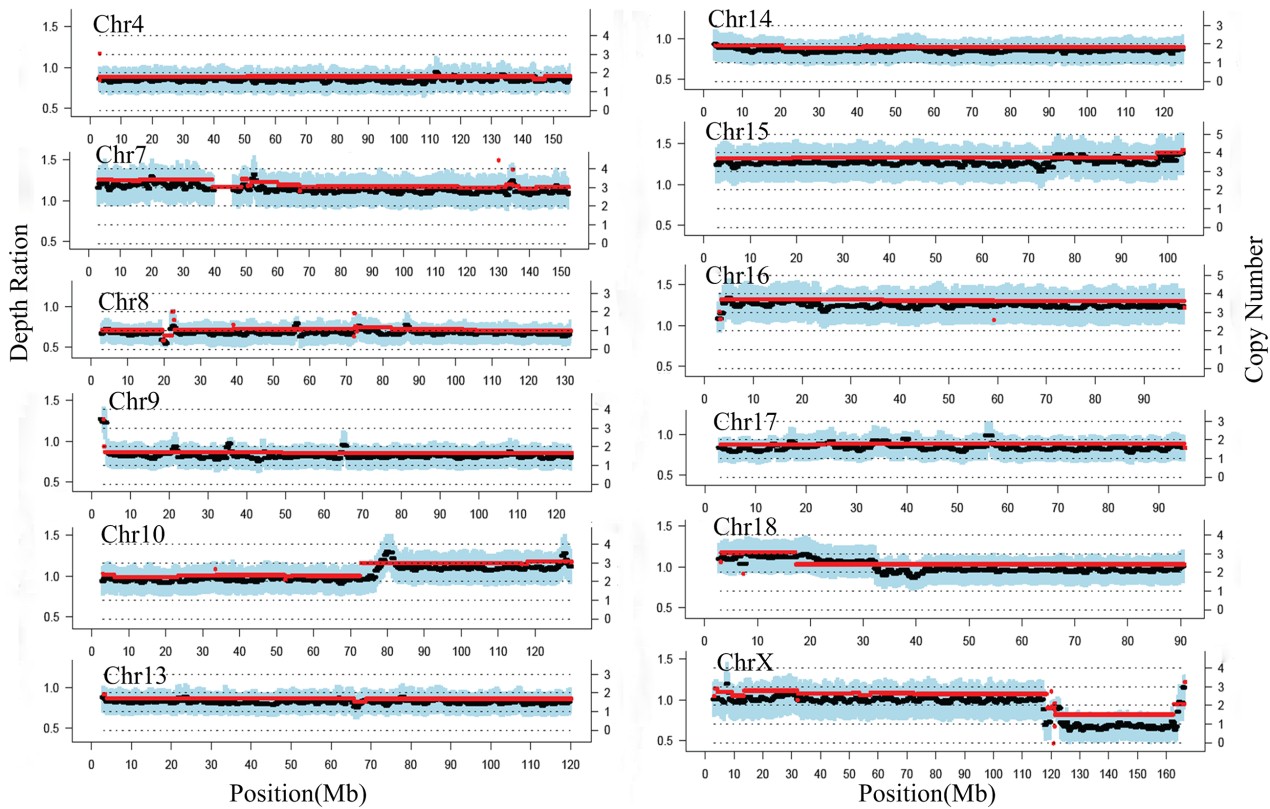

Supplement: S3 Fig — CNVs were called with Sequenza, which is based on the read depth of the whole genome sequencing data compared with the normal control. (JPG) [file pone.0187551.s004.jpg]

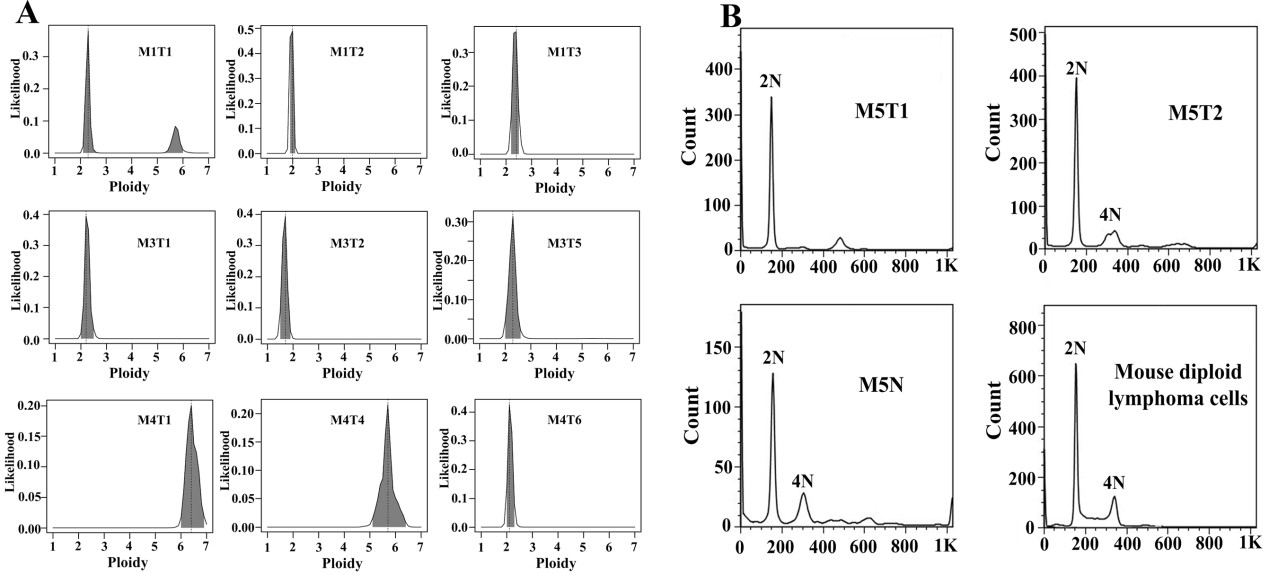

Supplement: S4 Fig — (A) Estimated karyotype of tumor cells based on sequencing data, and (B) karyotype of tumor cells determined by flow cytometry. (JPG) [file pone.0187551.s005.jpg]
